# Non-alcoholic fatty liver disease and dairy products consumption: Results from FASA Persian cohort study

**DOI:** 10.3389/fnut.2022.962834

**Published:** 2022-09-09

**Authors:** Zahra Keshavarz, Mehran Rahimlou, Mojtaba Farjam, Reza Homayounfar, Mahmoud Khodadost, Ashkan Abdollahi, Reza Tabrizi

**Affiliations:** ^1^Noncommunicable Diseases Research Center, Fasa University of Medical Sciences, Fasa, Iran; ^2^Department of Nutrition, School of Medicine, Zanjan University of Medical Sciences, Zanjan, Iran; ^3^National Nutrition and Food Technology Research Institute, Faculty of Nutrition Sciences and Food Technology, Shahid Beheshti University of Medical Sciences, Tehran, Iran; ^4^Department of Public Health, School of Health, Larestan University of Medical Sciences, Larestan, Iran; ^5^Student Research Committee, Shiraz University of Medical Sciences, Shiraz, Iran; ^6^Health Policy Research Center, Institute of Health, Shiraz University of Medical Sciences, Shiraz, Iran

**Keywords:** nonalcoholic fatty liver diseases, dairy, yogurt, cheese, cohort

## Abstract

**Background/objectives:**

There are limited data on the association between dairy products consumption and nonalcoholic fatty liver disease (NAFLD). This study was conducted to evaluate the association between total intake of different dairy products and fatty liver index (FLI), a marker of subclinical fatty liver.

**Methods:**

A total of 7,540 adults were included in this population-based cohort study. Dairy products consumption was evaluated by a validated interview questionnaire for food intake frequency. The FLI was calculated using the standard formula. Liver enzyme levels, lipid profiles, glycemic profiles and demographic characteristics were recorded for all participants. Univariate and multiple logistic regression models were used to respectively assess the mean percentage difference of mean FLI and odds ratios (ORs) for subclinical NAFLD across quantiles of dairy consumption.

**Results:**

The mean age of all participants was 48.81 ± 9.631 years. FLI measurements for men and women were 26.71 ± 23.39 and 39.99 ± 26.64 respectively, which was significantly higher in women (*P* < 0.05). Multiple logistic regression analysis demonstrated that the amount of milk consumption was an independent preventive predictor of FLI (OR = 0.96; 95% CI: 0.94–0.99), conversely, it did not predict higher levels of liver enzymes. In term of cheese intake, participants in the third tertile of cheese intake had significantly lower FLI than lower tertiles (*P* = 0.01). However, there wasn't any significant association between cheese intake and the odds of FLI in the multivariate model (*P* > 0.05). We didn't find any significant association between yogurt consumption and NAFLD indicators (*P* > 0.05).

**Conclusion:**

Higher milk consumption was inversely associated with FLI. However, there wasn't any significant association between other types of dairy products and NAFLD indicators.

## Introduction

Nonalcoholic Fatty Liver Disease (NAFLD) is a clinical histopathological condition in which triglyceride accumulates in the liver cells of patients with little or no history of alcohol consumption (1). This disease usually happens when fat accumulation reaches 5–10% of the liver's weight. NAFLD has a wide histological spectrum ranging from simple steatosis to non-alcoholic steatohepatitis (NASH) and more severe complications like cirrhosis or liver fibrosis (2, 3).

The total Prevalence of NAFLD based on a Meta-analytic study from 22 countries, has been reported as 25.24 % (4). In Asia, the number of patients with NAFLD is gradually increasing (5) due to lifestyle changes (high-fat diet, low physical activity, central obesity, and type 2 diabetes mellitus), which is comparable to Western countries (6).

NAFLD can lead to cirrhosis, cardiovascular disease, type 2 diabetes mellitus, and chronic kidney disease if left untreated (7).

Today, various studies have shown that improving the microbial profile of the intestine plays an important role in the prevention and treatment of NAFLD by reducing oxidative stress and inflammation, as well as decreasing triglycerides (TG) accumulation in the liver (8–12). As a result, the chance of hepatic steatosis will decrease. Dairy products are widely included in daily diet due to their high nutrition contents of protein, fat, minerals, and vitamins (13). Currently, it is recommended to limit the consumption of dairy products due to high levels of saturated fat and cholesterol, in order for reducing the risk of cardiovascular disease. However, a cross-sectional analysis of the Oslo Health Study has shown that increasing the frequency of consumption of some dairy products, such as cheese, is positively associated with improved serum concentrations of high-density lipoprotein (HDL) and is inversely related to serum triglyceride levels (14). Moreover, the consumption of some dairy products, especially those fortified with probiotics, appears to reduce the level of low-density lipoprotein (LDL) cholesterol in human intervention studies (15).

This study aims to focus on the relevance of dairy products consumption with NAFLD and fatty liver index in a large-population cohort in Fasa, Iran.

## Patients and methods

### Study population

In a population-based cohort, at least 10,000 people within the age range of 35–70 years old from Sheshdeh, the suburb of Fasa city and its 24 satellite villages were recruited. A detailed demographic, socioeconomic, anthropometric, nutrition, and medical history was obtained for each individual besides limited physical examinations and determination of physical activity and sleep patterns supplemented by body composition and electrocardiographic records (16). Participants older than 35 years with one of the following conditions were included in the study: alanine aminotransferase level >30 U/L, Gamma-glutamyltransferase >35 U/L.

Individuals with evidence of alcohol consumption of more than 20 grams per day, hepatitis, drug-induced liver disease, Wilson's disease, hereditary hemochromatosis, encephalopathy, or variceal bleeding were excluded from the study. Routine laboratory assessments were done, and a comprehensive biobank was compiled for future biological investigations. All data were stored online using a dedicated software.

## Measurement of clinical parameters and biochemical analysis

Fatty liver index (FLI) is a standard predictor of hepatic steatosis severity. The following formula was designed and validated by Bogoni et al. for calculating FLI using laboratory and anthropometric data, including gamma glutamyl transferase concentration, triglyceride, Body mass index and waist circumference (17).

FLI = (e 0.953 × loge (TG) + 0.139 × BMI + 0.718 × loge (GGT) + 0.053 × WC – 15.745) / (1 + e 0.953 × loge (TG) + 0.139 × BMI + 0.718 × loge (GGT) + 0.053 × WC – 15.745) × 100

To calculate the amount of dairy intake, a validated food frequency questionnaire (FFQ) was used. Different dairy products consumption was recorded separately according to the amount of daily, weekly, monthly and annual intake units. The questionnaire included 100 items with a specified serving size, 7 response frequency categories from “almost never eat” to “twice or more per day” for foods, and 8 response frequency categories from “almost never drink” to “Four or more times a day” for beverages. Total daily energy and nutrient intake are calculated based on the latest available table of Iranian food composition. Frequency of consumption of dairy products (milk, yogurt, cheese, doogh, curd and cream and any other dairy products) in the last month was assessed using 7 categories of answers as follows: Almost never, less than once a week, once a week, 2–3 times a week, 4–6 times a week, once a day and twice a day. Detailed information about the design, foods included and the validity of this questionnaire has been reported elsewhere (18). Previous validation studies of this FFQ designed specifically for Iranian adults revealed good correlation between dietary intakes assessed by a similar FFQ and multiple days of 24-h dietary recalls (18, 19).

### Statistical analysis

The characteristics of the participants across this study were showed in quartiles of FLI. Continuous variables were presented as mean ± SD and categorical variables as frequency and percentages (%). Data were analyzed using SPSS (version 18; SPSS Inc., Chicago, IL, USA) by running the student's *t*-test and one way ANOVA, and chi-squared tests. Odds ratios (ORs) and corresponding 95% confidence intervals (CIs) were estimated from univariate and multiple logistic regression for being classified with an undesirable level of FLI, ALT, AST and GGT and increasing quartile groups (Q1–Q4) for intake of dairy products. Multiple logistic regression models using backward elimination method were applied, adjusted for age, energy, physical activity, BMI, vitamin D levels, selenium levels, smoking status, hypertension, hypercholesterolemia, and diabetes mellitus, and for each relevant food items. Statistical tests were two-sided and *P* < 0.05 was considered statistically significant.

## Results

### Characteristics of study participants by sex and age

Characteristics of the study participants are presented in the Table 1. Data from 7,540 participants (59% female) were included in the final analysis. The mean age of all participants in this study was 48.81 ± 9.631 years. BMI was significantly higher in females as compared with males (26.63 ± 4.79 kg/m^2^ vs. 23.27 ± 4.17 kg/m^2^; *P* < 0.001). FLI was higher in females than males (39.991 ± 26.64 vs. 26.708 ± 23.39; *P* < 0.05). Also, serum levels of HDL, TC, LDL, FBS, SBP and DBP were significantly higher in females than males (*P* < 0.001). Dietary intakes of study participants across quartile of FLI score are presented in Table 2. A greater FLI score was significantly associated with the higher intake of carbohydrates, fruits, vegetables, meats, beans, dairy, vitamin D and selenium (*P* < 0.05).

**Table 1:** Baseline characteristics of study participants.

**Variables**	**Total (*n =* 7,540)**	**Men (*n =* 3,048)**	**Women (*n =* 4,492)**	***P*** **-value**
Age	48.81 ± 9.631	49.48 ± 9.666	48.36 ± 9.581	<0.001
BMI (kg/m2)	25.27 ± 4.84	23.27 ± 4.17	26.63 ± 4.79	<0.001
Waist circumference (cm)	92.274 ± 11.8734	87.483 ± 10.7930	95.526 ± 11.4660	<0.001
Current smoker (%)	1,985 (26.3 %)	1,770 (58.1 %)	215 (4.8 %)	<0.001
Physical activities (Met-h/day)	41.379 ± 11.078	45.622 ± 14.346	38.499 ± 6.772	<0.001
Total cholesterol (mg/dl)	182.70 ± 37.808	174.03 ± 34.775	188.58 ± 38.645	<0.001
Triglycerides (mg/dl)	122.89 ± 71.818	121.63 ± 72.313	123.74 ± 71.476	0.210
HDL-cholesterol (mg/dl)	51.66 ± 16.026	47.64 ± 14.525	54.39 ± 16.419	<0.001
LDL-cholesterol (mg/dl)	106.41 ± 31.882	101.99 ± 29.820	109.41 ± 32.874	<0.001
Fasting glucose (mg/dl)	90.90 ± 25.815	88.66 ± 21.220	92.42 ± 28.417	<0.001
AST (U/l)	20.20 ± 4.584	21.07 ± 4.633	19.60 ± 4.454	<0.001
ALT (U/l)	18.02 ± 5.385	18.65 ± 5.677	17.59 ± 5.133	<0.001
GGT (U/l)	17.08 ± 7.882	18.58 ± 8.311	16.07 ± 7.408	<0.001
ALP (U/I)	203.27 ± 62.184	205.58 ± 58.175	201.71 ± 64.723	0.008
SBP (mmHg)	110.69 ± 18.655	109.19 ± 17.692	111.70 ± 19.216	<0.001
DBP (mmHg)	74.08 ± 11.916	73.15 ± 11.654	74.72 ± 12.050	<0.001
FLI	34.621 ± 26.202	26.708 ± 23.398	39.991 ± 26.641	<0.001

BMI, body mass index; HDL, high-density lipoprotein; LDL, low-density lipoprotein; AST, aspartate aminotransferase; ALT, alanine aminotransferase; GGT, gamma-glutamyl transferase; ALP, alkaline phosphatase; SBP, systolic blood pressure; DBP, diastolic blood pressure; FLI; fatty liver index.

Data are mean ± standard deviation or as n (%), The data analyzed using Student's t-test or chi-square test.

**Table 2:** Participant characteristic and dietary intake according to FLI quartile.

	**Total FLI, quartiles**				
	**Quartile 1 (*n =* 1,885)**	**Quartile 2 (*n =* 1,884)**	**Quartile 3 (*n =* 1886)**	**Quartile 4 (*n =* 1885)**	***P*** **-value**
FLI	6.395 ± 2.852	19.195 ± 4.799	40.314 ± 7.263	72.569 ± 12.454	<0.001
BMI, kg/m^2^	19.999 ± 2.211	23.630 ± 2.135	26.435 ± 2.225	31.042 ± 3.783	<0.001
Age	47.49 ± 9.469	48.94 ± 9.948	49.16 ± 9.812	49.65 ± 9.147	<0.001
Smoking status, %	779 (41.4%)	490 (26.0%)	407 (21.6%)	309 (16.4%)	<0.001
Diabetes, %	81 (4.3%)	173 (9.2%)	237 (12.6%)	339 (18.0%)	<0.001
Hypertension, %	147 (7.8%)	301 (16.0%)	410 (21.7%)	651 (34.5%)	<0.001
Hypercholesterolemia, %	153 (8.1%)	249 (13.2%)	327 (17.3%)	455 (24.1%)	<0.001
physical activity (Met-h/day)	44.523 ± 12.922	42.266 ± 11.873	40.320 ± 9.875	38.407 ± 8.059	<0.001
Energy, kcal	2,949.230 ± 1,159.784	2,976.097 ± 1,114.890	2,886.361 ± 1,121.060	2,909.666 ± 1,145.891	0.071
Protein, g	94.558 ± 40.842	92.953 ± 37.827	92.357 ± 38.295	92.013 ± 38.633	0.191
Carbohydrate, g	501.27 ± 204.83	490.19 ± 197.97	491.97 ± 206.10	470.85 ± 188.48	<0.001
Fruit, g/d	353.096 ± 316.174	379.641 ± 302.302	399.193 ± 318.931	423.674 ± 335.956	<0.001
Vegetables, g/d	597.414 ± 338.962	636.174 ± 331.090	664.631 ± 350.200	717.518 ± 403.156	<0.001
Grains, g/d	372.769 ± 204.430	381.838 ± 212.351	379.374 ± 211.520	382.936 ± 211.981	0.447
Meats and beans g/d	150.558 ± 91.025	145.492 ± 83.309	140.905 ± 80.312	140.087 ± 83.842	<0.001
Dairy, g/d	201.501 ± 181.891	200.343 ± 162.967	209.012 ± 177.231	223.612 ± 184.515	<0.001
Milk, g/d	41.894 ± 64.497	41.918 ± 63.989	42.078 ± 79.580	41.478 ± 69.028	0.994
Cheese, g/d	18.448 ± 19.933	16.865 ± 16.244	17.243 ± 17.721	19.509 ± 21.458	<0.001
Yogurt, g/d	88.754 ± 92.848	89.789 ± 87.997	95.806 ± 94.337	103.108 ± 100.118	<0.001
Calcium, mg	1,417.222 ± 712.958	1,393.980 ± 657.089	1,400.386 ± 671.894	1,398.778 ± 663.375	0.737
Riboflavin, mg	2.453 ± 1.075	2.399 ± 1.002	2.378 ± 0.992	2.393 ± 1.005	0.120
Vitamin D, IUs	1.435 ± 1.178	1.412 ± 1.030	1.348 ± 1.016	1.345 ± 0.975	0.014
Vitamin E, mg	8.388 ± 4.124	8.533 ± 4.096	8.299 ± 3.781	8.484 ± 4.019	0.282
Selenium	152.895 ± 77.523	147.591 ± 73.298	144.941 ± 73.542	142.119 ± 72.438	<0.001

FLI; fatty liver index; BMI, body mass index.

Data are mean ± standard deviation or as n (%), The data analyzed using Student's t-test or chi-square test.

### Association of fatty liver index with dairy products

Crude and multiple-adjusted models for mean intake of dairy intake across quartiles of FLI are shown in Table 3. After controlling for potential confounders, participants in the highest quartile of FLI didn't have higher odds for dairy products intake [(OR = 1.00; 95% CI: 0.99, 1.00; *P* = 0.084) for males and (OR = 1.00; 95% CI: 0.99, 1.01; *P* = 0.306) for females]. Also, both females and males in the highest quartile of FLI didn't have higher odds for consumption of milk, yogurt, cheese, doogh, curd and cream (*P* > 0.05). We also examined the association between ALT and AST quartiles and odds of dairy consumption. The results showed that individuals in the highest ALT quartile did not have odds of more dairy products consumption. Participants in the highest quartile of AST had higher odds only for cream intake (OR= 0.97; 95% CI: 0.95, 0.99; P_trend_ = 0.011). There wasn't any significant association between other dairy products and ALT and AST quartiles (*P* > 0.05).

**Table 3:** Odds ratio (95% CI) from multiple logistic regression by dairy food type.

	**Sex**	**Crude model**					**Adjusted model** ^a^				
**FLI**		**Q1 (*M =* 1,112, *W =* 773)**	**Q2 (*M =* 818, *W =* 1,066)**	**Q3 (*M =* 649, *W =* 1,237)**	**Q4 (*M =* 469, *W =* 1,416)**	***P*** **-trend**	**Q1 (*M =* 1,112, *W =* 773)**	**Q2 (*M =* 818, *W =* 1,066)**	**Q3 (*M =* 649, *W =* 1,237)**	**Q4 (*M =* 469, *W =* 1,416)**	***P*** **-trend**
Dairy products	M	1 (Ref)	1.00 (0.99–1.00) *P =* 0.611	1.00 (1.00–1.00) *P =* 0.501	1.00 (1.00–1.00) *P =* 0.084	0.201	1 (Ref)	1.00 (0.99–1.00) *P =* 0.350	1.00 (0.99–1.00) *P =* 0.686	1.00 (0.99–1.00) *P =* 0.701	0.811
	W	1 (Ref)	1.00 (1.00–1.00) *P =* 0.036	1.00 (1.00–1.00) *P =* 0.000	1.00 (1.00–1.00) *P =* 0.000	<0.001	1 (Ref)	1.00 (1.00–1.00) *P =* 0.291	1.00 (0.99–1.00) *P =* 0.447	1.00 (0.99–1.01) *P =* 0.306	0.671
Milk	M	1 (Ref)	1.00 (0.99–1.00) *P =* 0.855	1.00 (0.99–1.00) *P =* 0.896	0.99 (0.99–1.00) *P =* 0.316	0.777	1 (Ref)	1.00 (0.99–1.00) *P =* 0.953	1.00 (0.99–1.00) *P =* 0.813	0.99 (0.99–1.00) *P =* 0.188	0.404
	W	1 (Ref)	1.00 (0.99–1.00) *P =* 0.313	1.00 (1.00–1.00) *P =* 0.192	1.00 (1.00–1.00) *P =* 0.113	0.421	1 (Ref)	1.00 (1.00–1.01) *P =* 0.119	1.00 (0.99–1.00) *P =* 0.263	1.00 (0.99–1.01) *P =* 0.346	0.452
Cheese	M	1 (Ref)	0.99 (0.99–0.99) *P =* 0.030	0.99 (0.98–0.99) *P =* 0.028	1.00 (0.99–1.01) *P =* 0.318	0.006	1 (Ref)	0.99 (0.99–1.01) *P =* 0.291	0.99 (0.98–1.01) *P =* 0.215	1.01 (0.99–1.02) *P =* 0.274	0.224
	W	1 (Ref)	1.00 (0.99–1.01) *P =* 0.991	1.00 (0.99–1.01) *P =* 0.166	1.01 (1.00–1.02) *P =* 0.000	<0.001	1 (Ref)	0.99 (0.98–1.01) *P =* 0.568	1.00 (0.99–1.01) *P =* 0.972	1.01 (0.99–1.02) *P =* 0.310	0.126
Yogurt	M	1 (Ref)	1.00 (0.99–1.00) *P =* 0.603	1.00 (1.00–1.00) *P =* 0.057	1.00 (1.00–1.00) *P =* 0.012	0.047	1 (Ref)	1.00 (0.99–1.00) *P =* 0.593	1.00 (0.99–1.00) *P =* 0.991	1.00 (0.99–1.00) *P =* 0.861	0.890
	W	1 (Ref)	1.00 (1.00–1.00) *P =* 0.182	1.00 (1.00–1.00) *P =* 0.001	1.00 (1.00–1.01) *P =* 0.000	<0.001	1 (Ref)	0.99 (0.99–1.00) *P =* 0.542	0.99 (0.99–1.00) *P =* 0.498	1.00 (0.99–1.00) *P =* 0.838	0.798
Dough	M	1 (Ref)	1.00 (0.99–1.00) *P =* 0.437	1.00 (0.99–1.00) *P =* 0.636	1.00 (1.00–1.00) *P =* 0.139	0.229	1 (Ref)	0.99 (0.99–1.00) *P =* 0.321	1.00 (0.99–1.00) *P =* 0.937	1.00 (0.99–1.00) *P =* 0.972	0.667
	W	1 (Ref)	1.00 (1.00–1.00) *P =* 0.182	1.00 (1.00–1.00) *P =* 0.001	1.00 (1.00–1.01) *P =* 0.000	<0.001	1 (Ref)	1.00 (1.00–1.01) *P =* 0.088	1.00 (0.99–1.00) *P =* 0.144	1.00 (0.99–1.00) *P =* 0.142	0.384
Curd	M	1 (Ref)	0.98 (0.96–1.00) *P =* 0.089	0.98 (0.96–1.01) *P =* 0.129	1.01 (0.98–1.03) *P =* 0.404	0.058	1 (Ref)	0.97 (0.95–1.01) *P =* 0.146	0.98 (0.94–1.02) *P =* 0.389	0.99 (0.95–1.04) *P =* 0.878	0.391
	W	1 (Ref)	0.99 (0.97–1.03) *P =* 0.447	0.99 (0.97–1.01) *P =* 0.461	1.00 (0.98–1.02) *P =* 0.985	0.710	1 (Ref)	0.98 (0.95–1.01) *P =* 0.295	0.99 (0.95–1.03) *P =* 0.492	0.95 (0.89–1.01) *P =* 0.101	0.262
Cream	M	1 (Ref)	0.97 (0.95–0.99) *P =* 0.042	0.98 (0.96–1.01) *P =* 0.125	0.96 (0.93–0.99) *P =* 0.022	0.029	1 (Ref)	1.01 (0.96–1.06) *P =* 0.693	1.02 (0.97–1.07) *P =* 0.484	1.02 (0.97–1.08) *P =* 0.423	0.791
	W	1 (Ref)	1.00 (0.98–1.03) *P =* 0.754	1.01 (0.98–1.03) *P =* 0.682	0.99 (0.97–1.02) *P =* 0.932	0.925	1 (Ref)	0.99 (0.96–1.02) *P =* 0.592	0.98 (0.94–1.03) *P =* 0.460	0.99 (0.95–1.04) *P =* 0.962	0.622
Dairy products	M	1 (Ref)	1.00 (1.00–1.00) *P =* 0.171	1.00 (1.00–1.00) *P =* 0.632	1.00 (1.00–1.00) *P =* 0.002	0.006	1 (Ref)	1.00 (0.99–1.00) *P =* 0.873	1.00 (0.99–1.00) *P =* 0.153	1.00 (1.00–1.00) *P =* 0.335	0.063
	W	1 (Ref)	1.00 (1.00–1.00) *P =* 0.436	1.00 (1.00–1.00) *P =* 0.216	1.00 (1.00–1.00) *P =* 0.178	0.514	1 (Ref)	1.00 (0.99–1.00) *P =* 0.847	1.00 (0.99–1.00) *P =* 0.665	1.00 (0.99–1.00) *P =* 0.729	0.975
Milk	M	1 (Ref)	1.00 (0.99–1.00) *P =* 0.639	0.99 (0.99–1.00) *P =* 0.407	1.00 (0.99–1.00) *P =* 0.397	0.318	1 (Ref)	0.99 (0.99–1.00) *P =* 0.134	0.99 (0.99–1.00) *P =* 0.054	1.00 (0.99–1.00) *P =* 0.703	0.107
	W	1 (Ref)	1.00 (0.99–1.00) *P =* 0.784	1.00 (0.99–1.00) *P =* 0.934	1.00 (0.99–1.00) *P =* 0.293	0.669	1 (Ref)	1.00 (0.99–1.00) *P =* 0.971	1.00 (0.99–1.00) *P =* 0.551	1.00 (0.99–1.00) *P =* 0.488	0.641
Cheese	M	1 (Ref)	1.00 (0.99–1.01) *P =* 0.099	0.99 (0.99–1.01) *P =* 0.775	1.01 (1.00–1.01) *P =* 0.034	0.030	1 (Ref)	1.00 (0.99–1.01) *P =* 0.183	0.99 (0.99–1.00) *P =* 0.417	1.00 (0.99–1.01) *P =* 0.164	0.053
	W	1 (Ref)	0.99 (0.99–1.00) *P =* 0.576	1.00 (0.99–1.00) *P =* 0.697	1.01 (1.00–1.01) *P =* 0.035	0.052	1 (Ref)	0.99 (0.99–1.00) *P =* 0.554	1.00 (0.99–1.01) *P =* 0.948	1.00 (0.99–1.01) *P =* 0.086	0.122
Yogurt	M	1 (Ref)	1.00 (0.99–1.00) *P =* 0.591	1.00 (0.99–1.00) *P =* 0.747	1.00 (1.00–1.00) *P =* 0.068	0.236	1 (Ref)	1.00 (0.99–1.01) *P =* 0.518	0.99 (0.99–1.00) *P =* 0.234	1.00 (0.99–1.00) *P =* 0.982	0.489
	W	1 (Ref)	1.00 (1.00–1.00) *P =* 0.236	1.00 (1.00–1.00) *P =* 0.173	1.00 (0.99–1.00) *P =* 0.847	0.438	1 (Ref)	1.00 (0.99–1.00) *P =* 0.815	1.00 (0.99–1.00) *P =* 0.811	0.99 (0.99–1.00) *P =* 0.139	0.324
Dough	M	1 (Ref)	1.00 (1.00–1.01) *P =* 0.005	1.00 (1.00–1.01) *P =* 0.035	1.00 (1.00–1.01) *P =* 0.000	0.001	1 (Ref)	1.00 (1.00–1.01) *P =* 0.135	1.00 (0.99–1.00) *P =* 0.529	1.00 (1.00–1.01) *P =* 0.098	0.077
	W	1 (Ref)	1.00 (0.99–1.00) *P =* 0.789	1.00 (1.00–1.00) *P =* 0.240	1.00 (1.00–1.00) *P =* 0.241	0.522	1 (Ref)	1.00 (0.99–1.00) *P =* 0.657	1.00 (0.99–1.00) *P =* 0.981	1.00 (0.99–1.00) *P =* 0.916	0.968
Curd	M	1 (Ref)	0.99 (0.97–1.01) *P =* 0.694	0.99 (0.97–1.02) *P =* 0.538	1.01 (0.98–1.02) *P =* 0.475	0.508	1 (Ref)	0.99 (0.97–1.02) *P =* 0.480	0.98 (0.96–1.01) *P =* 0.352	1.00 (0.98–1.02) *P =* 0.769	0.481
	W	1 (Ref)	0.99 (0.97–1.01) *P =* 0.334	0.99 (0.98–1.01) *P =* 0.613	1.00 (0.98–1.02) *P =* 0.952	0.723	1 (Ref)	0.98 (0.97–1.01) *P =* 0.220	0.99 (0.97–1.01) *P =* 0.331	0.99 (0.97–1.02) *P =* 0.770	0.579
Cream	M	1 (Ref)	0.98 (0.96–1.01) *P =* 0.254	0.98 (0.96–1.01) *P =* 0.190	0.99 (0.96–1.01) *P =* 0.267	0.522	1 (Ref)	0.98 (0.96–1.01) *P =* 0.322	0.98 (0.96–1.01) *P =* 0.252	0.99 (0.97–1.01) *P =* 0.436	0.657
	W	1 (Ref)	1.01 (0.98–1.02) *P =* 0.570	0.99 (0.97–1.02) *P =* 0.945	1.00 (0.98–1.02) *P =* 0.906	0.912	1 (Ref)	1.01 (0.98–1.03) *P =* 0.464	1.00 (0.97–1.02) *P =* 0.872	1.01 (0.98–1.03) *P =* 0.580	0.846
Dairy products	M	1 (Ref)	1.00 (0.99–1.00) *P =* 0.439	1.00 (0.99–1.00) *P =* 0.633	1.00 (1.00–1.00) *P =* 0.318	0.191	1 (Ref)	1.00 (0.99–1.00) *P =* 0.273	1.00 (0.99–1.00) *P =* 0.163	1.00 (0.99–1.00) *P =* 0.985	0.280
	W	1 (Ref)	1.00 (0.99–1.00) *P =* 0.159	1.00 (0.99–1.00) *P =* 0.520	1.00 (0.99–1.00) *P =* 0.132	0.376	1 (Ref)	1.00 (0.99–1.00) *P =* 0.215	1.00 (0.99–1.00) *P =* 0.289	1.00 (0.99–1.00) *P =* 0.097	0.366
Milk	M	1 (Ref)	0.99 (0.99–1.00) *P =* 0.182	1.00 (0.99–1.00) *P =* 0.631	1.00 (0.99–1.00) *P =* 0.587	0.199	1 (Ref)	0.99 (0.99–1.00) *P =* 0.137	0.99 (0.99–1.00) *P =* 0.287	1.00 (0.99–1.00) *P =* 0.866	0.323
	W	1 (Ref)	1.00 (0.99–1.00) *P =* 0.658	1.00 (0.99–1.00) *P =* 0.558	1.00 (0.99–1.00) *P =* 0.937	0.803	1 (Ref)	1.00 (0.99–1.00) *P =* 0.708	1.00 (0.99–1.00) *P =* 0.712	1.00 (0.99–1.00) *P =* 0.831	0.897
Cheese	M	1 (Ref)	1.00 (0.99–1.01) *P =* 0.621	0.99 (0.99–1.00) *P =* 0.761	1.00 (0.99–1.01) *P =* 0.239	0.362	1 (Ref)	1.00 (0.99–1.01) *P =* 0.747	0.99 (0.99–1.00) *P =* 0.511	1.00 (0.99–1.01) *P =* 0.484	0.450
	W	1 (Ref)	0.99 (0.99–1.00) *P =* 0.099	0.99 (0.99–1.00) *P =* 0.687	0.99 (0.99–1.00) *P =* 0.104	0.225	1 (Ref)	0.99 (0.99–1.00) *P =* 0.215	1.00 (0.99–1.00) *P =* 0.877	0.99 (0.99–1.01) *P =* 0.476	0.496
Yogurt	M	1 (Ref)	1.00 (0.99–1.00) *P =* 0.886	1.00 (0.99–1.00) *P =* 0.417	1.00 (0.99–1.00) *P =* 0.551	0.454	1 (Ref)	1.00 (0.99–1.00) *P =* 0.964	0.99 (0.99–1.00) *P =* 0.141	1.00 (0.99–1.00) *P =* 0.917	0.297
	W	1 (Ref)	1.00 (0.99–1.00) *P =* 0.571	1.00 (0.99–1.00) *P =* 0.772	0.99 (0.99–1.00) *P =* 0.057	0.180	1 (Ref)	1.00 (0.99–1.00) *P =* 0.713	1.00 (0.99–1.00) *P =* 0.584	0.99 (0.99–1.00) *P =* 0.050	0.199
Dough	M	1 (Ref)	0.99 (0.99–1.00) *P =* 0.313	1.00 (0.99–1.00) *P =* 0.498	1.00 (0.99–1.00) *P =* 0.275	0.109	1 (Ref)	0.99 (0.98–1.00) *P =* 0.204	1.00 (0.99–1.00) *P =* 0.963	1.00 (0.99–1.00) *P =* 0.708	0.296
	W	1 (Ref)	0.99 (0.99–1.00) *P =* 0.118	0.99 (0.99–1.00) *P =* 0.128	1.00 (0.99–1.00) *P =* 0.655	0.294	1 (Ref)	0.99 (0.99–1.00) *P =* 0.123	0.99 (0.99–1.00) *P =* 0.055	1.00 (0.99–1.00) *P =* 0.378	0.188
Curd	M	1 (Ref)	1.00 (0.97–1.02) *P =* 0.988	0.99 (0.96–1.01) *P =* 0.422	0.99 (0.97–1.01) *P =* 0.556	0.784	1 (Ref)	0.99 (0.97–1.02) *P =* 0.876	0.98 (0.96–1.01) *P =* 0.277	0.99 (0.96–1.01) *P =* 0.375	0.612
	W	1 (Ref)	0.99 (0.98–1.01) *P =* 0.756	0.98 (0.96–1.00) *P =* 0.159	1.00 (0.98–1.02) *P =* 0.984	0.480	1 (Ref)	0.99 (0.98–1.01) *P =* 0.744	0.98 (0.95–1.00) *P =* 0.057	0.99 (0.97–1.01) *P =* 0.576	0.236
Cream	M	1 (Ref)	0.98 (0.97–1.01) *P =* 0.329	0.96 (0.93–0.98) *P =* 0.005	0.97 (0.95–0.99) *P =* 0.031	0.017	1 (Ref)	0.98 (0.96–1.01) *P =* 0.293	0.96 (0.94–0.98) *P =* 0.004	0.97 (0.95–0.99) *P =* 0.020	0.011
	W	1 (Ref)	1.01 (0.98–1.03) *P =* 0.372	1.01 (0.98–1.02) *P =* 0.960	1.01 (0.98–1.03) *P =* 0.602	0.813	1 (Ref)	1.01 (0.98–1.04) *P =* 0.312	1.01 (0.98–1.03) *P =* 0.551	1.01 (0.98–1.03) *P =* 0.472	0.726

W, women; M, men.

Odds ratio (95% CI) are from multiple logistic regression for being classified with an undesirable level of FLI, ALT, AST and GGT and increasing quintile groups (Q1–Q4) for intake of dairy products. Q1, lowest intake, is the reference category. Statistically significant p-values are in supers.

a **Multivariable-adjusted model adjusted** for age (years), energy (kcals/day), physical activity (minutes/week), BMI (kg/m2), Vitamin D levels, Selenium levels, smoking status (yes, no), hypertension (yes, no), hypercholesterolemia (yes, no), and diabetes (yes, no), and for each relevant food items.

The results of univariate and multiple analyses for determining independent association between fatty liver index and dairy consumption are shown in the Table 4. The mean FLI in the tertile 3 milk intake was 34.08 (95% CI: 32.99, 35.17) that wasn't significantly different from tertile 1 (*P* = 0.171). Mean liver enzymes did not differ significantly in different milk consumption tertiles (*P* = 0.08 for AST, *P* = 0.174 for ALT, and *P* = 0.491 for GGT).

**Table 4:** Univariate and multiple analyses to determine the independent association between fatty liver index and dairy consumption.

	**Milk**			
	**Tertile 1 (*n =* 2,952)**	**Tertile 2 (*n =* 2,368)**	**Tertile 3 (*n =* 2,220)**	
**Mean (95% CI)**				***P*** **–value ANOVA**
FLI	35.32 (34.35–36.29)	34.25 (33.23–35.27)	34.08 (32.99–35.17)	0.171
AST	20.07 (19.91–20.24)	20.20 (20.02–20.39)	20.35 (20.16–20.55)	0.088
ALT	17.96 (17.77–18.15)	17.92 (17.71–18.14)	18.20 (17.97–18.42)	0.174
GGT	17.21 (16.92–17.51)	16.97 (16.68–17.25)	17.03 (16.68–17.37)	0.491
**OR (95% CI)** ^a^				
FLI	1 (Ref)	0.99 (0.99–1.00)^0.230^	0.96 (0.94–0.99)^0.025^	
AST	1 (Ref)	1.00 (0.99–1.02)^0.608^	1.00 (0.99–1.02)^0.223^	
ALT	1 (Ref)	0.99 (0.98–1.01)^0.529^	1.00 (0.99–1.01)^0.826^	
GGT	1 (Ref)	0.99 (0.98–1.00)^0.260^	0.99 (0.99–1.00)^0.439^	
	**Cheese**			
	**Tertile 1 (*****n** =* **3,075)**	**Tertile 2 (*****n** =* **1,996)**	**Tertile 3 (*****n** =* **2,469)**	
**Mean (95% CI)**				***P*** **–value ANOVA**
FLI	35.55 (32.65–34.46)	35.73 (34.57–36.89)	35.04 (33.98–36.10)	0.010
AST	20.14 (19.98–20.30)	20.22 (20.03–20.42)	20.24 (20.06–20.43)	0.678
ALT	17.75 (17.56–17.94)	18.16 (17.93–18.40)	18.24 (18.02–18.45)	0.001
GGT	16.84 (16.59–17.08)	17.32 (16.96–17.67)	17.19 (16.85–17.54)	0.071
**OR (95% CI)** ^a^				
FLI	1 (Ref)	1.00 (0.99–1.01)^0.137^	1.00 (0.99–1.01)^0.936^	
AST	1 (Ref)	1.01 (0.99–1.02)^0.326^	1.00 (0.98–1.01)^0.850^	
ALT	1 (Ref)	1.01 (0.99–1.02)^0.119^	1.00 (0.99–1.01)^0.373^	
GGT	1 (Ref)	1.01 (0.99–1.01)^0.110^	1.00 (0.99–1.01)^0.598^	
	**Yogurt**			
	**Tertile 1 (*****n** =* **2,514)**	**Tertile 2 (*****n** =* **2,938)**	**Tertile 3 (*****n** =* **2,088)**	
**Mean (95% CI)**				***P*** **–value ANOVA**
FLI	32.73 (31.71–33.74)	34.75 (33.81–35.69)	36.70 (35.56–37.85)	0.000
AST	20.13 (19.96–20.31)	20.37 (20.20–20.54)	20.03 (19.84–20.22)	0.026
ALT	17.84 (17.63–18.06)	18.10 (17.90–18.30)	18.11 (17.88–18.34)	0.143
GGT	16.86 (16.58–17.13)	17.05 (16.75–17.35)	17.39 (17.03–17.74)	0.073
**OR (95% CI)** ^a^				
FLI	1 (Ref)	1.00 (0.99–1.00)^0.577^	1.00 (0.99–1.01)^0.879^	
AST	1 (Ref)	1.01 (0.99–1.02)^0.201^	0.98 (0.97–1.00)^0.073^	
ALT	1 (Ref)	0.99 (0.98–1.01)^0.815^	0.99 (0.97–1.00)^0.099^	
GGT	1 (Ref)	0.96 (1.00–0.99)^0.964^	1.00 (0.99–1.01)^0.523^	

a **Multivariable-adjusted model adjusted** for sex, age (years), energy (kcals/day), physical activity (minutes/week), BMI (kg/m2), Vitamin D levels, Selenium levels, smoking status (yes, no), hypertension (yes, no), hypercholesterolemia (yes, no), and diabetes (yes, no), and for each relevant food items.

The association between the amount of dairy consumption and severity of fatty liver disease is shown in Table 4. When controlled for confounding factors, multiple logistic regression analysis demonstrated that the amount of milk consumption was the independent preventive predictor of FLI (OR = 0.96; 95% CI: 0.94, 0.99; *P* = 0.025), but was not the predictors of higher levels of AST, ALT and GGT (*P* > 0.05). For the cheese intake, participants in the tertile 3 had significantly lower FLI than the lowest tertile 35.04 (95% CI: 33.98, 36.10; *P* = 0.01). Individuals in the highest tertile of cheese intake had higher levels of ALT than lowest tertile 18.24 mg/dl (95% CI: 18.02, 18.45; *P* = 0.001). However, there wasn't any significant association between cheese intake and odds of fatty liver index (*P* > 0.05).

Participants in the highest tertile of yogurt intake had significantly higher FLI scores 36.70 (95% CI: 35.56, 37.85; *P* < 0.001) and AST concentration 20.03 mg/dl (95% CI: 19.84, 20.22; *P* = 0.026). We did not find a significant association between yogurt consumption and fatty liver index or biomarkers (*P* > 0.05).

## Discussion

The results of this study clearly indicate that participants with higher FLI score consume more dairy, cheese and yogurt compared to the subjects with lower FLI. Moreover, there is no strong correlation between dairy consumption and fatty liver indicators and only milk consumption is a preventive indicator of FLI. Also, in our study, the consumption of dairy products in women had more protective effects against NAFLD than in men.

The impacts of dairy products on the amounts of liver-derived lipoproteins have been widely studied and discussed previously (20, 21). Several studies have looked at dietary factors, including dairy intake as the key interventional variable on NAFLD-related parameters in NAFLD patients. In some observational studies, researchers have reported an inverse association between low-fat dairy intake and the severity of NAFLD. Ferolla et al. in a cross-sectional study of 96 patients with NAFLD observed an inverse association between low fat dairy intake and NAFLD severity (22). Similar findings were found in another study (23). Conversely, it has been shown that some dietary regiments which contain adequate amounts of dairy products such as DASH (Dietary Approaches to Stop Hypertension) can have a positive effect on reducing the symptoms of NAFLD. Razavi Zade et al. conducted a clinical trial with 3 daily servings with low dairy products to evaluate the effects of DASH diet on the NAFLD severity and showed adherence to DASH eating pattern leads to a significant reduction in serum levels of ALT, AST, and insulin resistance (24). However, part of the positive effects of DASH diet on fatty liver can be attributed to high amounts of fibers and phytochemicals in the DASH diet (25).

In the present study, except for milk and FLI correlation, we did not observe any correlations between dairy products and fatty liver biomarkers. Contrary to our findings, in a 6-week randomized controlled trial, Dugan et al. found a significant reduction in ALT, AST, hepatic steatosis index, and mRNA expression of IL-6 and IL-1β in peripheral blood mononuclear cells (PBMCs), following the consumption of 3 daily servings of low-fat dairy (296 mL 1% milk, 170 g non-fat yogurt, 56.7 g 2% cheese) as compared with isocaloric carbohydrate-based control foods (26). A recent meta-analysis was done on the association between food groups and the risk of NAFLD which included three cross sectional studies and one case control study. The cross-sectional studies didn't find a significant correlation between dairy consumption and the likelihood of NAFLD. However, the case–control study showed a positive association between dairy product consumption and the possibility of NAFLD (27).

The diversity in the type of dairy products and their fat percentages may partly account for the difference observed in the results. Most previous studies that have reported significant changes on fatty liver related indicators following dairy consumption have examined the effect of low-fat dairy products, while in our study participants consumed both high-fat and low-fat dairy products. In fact, the diet of Iranian people especially those in rural areas is mostly based on high consumption of high-fat dairy products (28). Esmaillzadeh et al. studied 486 healthy Iranian women aged 40–60 years and found an inverse association between low fat dairy intake and C-reactive protein, IL-6 and soluble vascular cell adhesion molecule (29). Moreover, they reported an increase in serum amyloid A and soluble vascular cell adhesion molecule-1 among women with high fat dairy consumption (30). High-fat dairy products contain high levels of saturated fatty acids which play a key role in dyslipidemia, insulin resistance and inflammation, that altogether are major risk factors for NAFLD (31).

In contrast with our findings, Zhang et al. in a cross-sectional study of 24,389 adults found that participants with yogurt consumption more than 4 times per weeks had lower likelihood for NAFLD (32). Other similar studies have shown a preventative role for yogurt consumption in the development of other chronic diseases such as cardiovascular disease and type 2 diabetes (29, 33). Most studies that have shown the positive effect of yogurt consumption on liver enzymes have used a variety of probiotic yogurts (9, 34).

In our study, participants in the higher FLI quartile consumed significantly more yogurt than subjects in the lower quartile of FLI. The present study was a cross-sectional study and due to the nature of cross-sectional studies it is not possible to identify the causal relationship between two variables. However, it seems that this difference might be attributed to the fact that people who are at risk of NAFLD increase their yogurt consumption to get more probiotics because of the positive role of bacteria in yogurt against NAFLD, especially probiotic yogurt. The positive effect of yogurt on fatty liver has been attributed to the presence of various probiotic compounds in yogurt. Animal studies have shown that some bacterial strains, such as Lactobacillus, have the ability to reduce the inflammation caused by high levels of lipopolysaccharide (LPS) and subsequent hepatic toll-like receptor 4 (TLR4) activation in NAFLD patients (35). Additionally, part of the beneficial effects of dairy products, especially low-fat dairy products, might be due to high amounts of nutrients, especially magnesium, potassium, protein and calcium in dairy products, which in turn can increase the whole-body fat oxidation (36, 37).

In the present study, we conducted sex-specific analyses which showed that the consumption of dairy products in women had stronger protective effects against NAFLD than in men. While the exact mechanisms for this difference are unclear, several studies have reported a higher risk of NALFD in men and post-menopausal women than in pre-menopausal women (38, 39). Interestingly, experimental results suggest a protective role for estradiol in hepatic injuries by suppressing lipid accumulation and liver fibrosis (40). The presence of estrone (E1) and 17β-estradiol (E2) in raw whole cow's milk has been demonstrated (41). Dairy products have been estimated to account for up to 60% of estrogens in a German diet (42). Some studies have shown that the estrogen in dairy products may increase the concentration of estrogen in the serum (43).

The results of some studies contradicted with the findings of our study. Lee et al. in a study of 5171 adults showed higher dairy protein intake was significantly and inversely associated with the risk of incident NAFLD in men and women aged ≥50 years (44). They suggested that replacing macronutrients equivalent to carbohydrate intake with dairy protein intake may have contributed to lowering the risk of developing NAFLD (32, 44). It has also been suggested that some of the beneficial effects of dairy products in preventing NAFLD are related to the whey protein found in dairy products. Several human and animal studies have shown that whey protein or dairy products reduce weight and fat mass (45–47). Other mechanisms have been suggested for the beneficial effects of dairy products against NAFLD, some of which are related to insulin resistance. The population-based prospective Coronary Artery Risk Development in Young Adults study found an inverse association between frequency of dairy intake and development of insulin resistance syndrome (48). Also, a meta-analysis study showed that the consumption of dairy products, especially low-fat dairy products had a beneficial effect on Homeostatic Model Assessment for Insulin Resistance (HOMA-IR), waist circumference, and body weight (49).

This study has several important advantages including the large sample size, and extensive information on lifestyle and dietary factors, which allowed us to control for many potential confounders. In this study, we adjusted the results for several variables, especially BMI which is a crude measure of body fat and is associated with NAFLD and may negatively affect the accuracy of the results (32). On the other hand, the present study was not free of limitations. Due to the structure of the questionnaires used, we were not able to examine low-fat and high-fat dairy products separately. Also, because of the nature of questionnaires filled in interviews, there is always possibility of bias which can affect the accuracy of the results. Another limitation of the current study was the presence of some potential confounding factors such as diabetes, hyperlipidemia, and high blood pressure and their variability between FLI quartile. However, in the adjusted models, we adjusted the effects of these confounding factors. Finally, because of the difficulties in budget management and the high sample size, we were not able to assess the liver through imaging studies. Evaluation of hepatic steatosis and fibrosis could increase the accuracy of the results in future studies.

## Conclusion

In summary, this population-based cohort study didn't show any strong association between dairy products consumption and NAFLD indicators. However, a modest inverse correlation was observed between milk consumption and FLI. Therefore, based on the results of this study, consumption of appropriate amounts of milk, especially low-fat types (at least one unit of milk more than 5–6 times a week) can play a positive role in preventing NAFLD. The results of the present study seem to be in line with the existing recommendations for consumption of a healthy dietary pattern to prevent NAFLD.

## Data availability statement

The raw data supporting the conclusions of this article will be made available by the authors, without undue reservation.

## Ethics statement

The study protocol was approved by the Research Council and the Ethics Committee of Fasa University of Medical Sciences (IR.FUMS.REC.1400.085). The patients/participants provided their written informed consent to participate in this study.

## Author contributions

ZK, MF, RH, and RT make substantial contributions to conception, design, acquisition of data, analysis and interpretation of data, and drafting of this study. ZK, MR, AA, and MK participate in drafting the article or revising it. MF and RH gives final approval of the version to be submitted and any revised version. MF and RT are the guarantors of this work. All authors have read and approved the manuscript.

## Funding

This study was supported by the Deputy of Research and Technology of Fasa University of Medical Sciences, Fasa, Iran (No. 400067).

## Conflict of interest

The authors declare that the research was conducted in the absence of any commercial or financial relationships that could be construed as a potential conflict of interest.

## Publisher's note

All claims expressed in this article are solely those of the authors and do not necessarily represent those of their affiliated organizations, or those of the publisher, the editors and the reviewers. Any product that may be evaluated in this article, or claim that may be made by its manufacturer, is not guaranteed or endorsed by the publisher.

## Abbreviations

NAFLD: Nonalcoholic fatty liver disease
FLI: Fatty liver index
OR: Odds ratio
FFQ: Food frequency questionnaire
BMI: body mass index
HDL: high-density lipoprotein
LDL: low-density lipoprotein
AST: aspartate aminotransferase
ALT: alanine aminotransferase
GGT: gamma-glutamyl transferase
ALP: alkaline phosphatase
SBP: systolic blood pressure
DBP: diastolic blood pressure
ANOVA: One-way Analysis of Variance.
